# RNA editing analysis of ATP synthase genes in the cotton cytoplasmic male sterile line H276A

**DOI:** 10.1186/s40659-019-0212-0

**Published:** 2019-02-06

**Authors:** Xiangjun Kong, Dongmei Liu, Jie Zheng, Aziz Khan, Bin Li, Yong Diao, Ruiyang Zhou

**Affiliations:** 1 Key Laboratory of Plant Genetic and Breeding, College of Agriculture, Guangxi University, Nanning, 530005 People’s Republic of China; 2 Shangqiu Normal University, Shangqiu, 476000 Henan People’s Republic of China

**Keywords:** Cotton, Cytoplasmic male sterility, ATP synthase gene, Molecular marker, RNA editing

## Abstract

**Background:**

Pollen development is an energy-consuming process that particularly occurs during meiosis. Low levels of adenosine triphosphate (ATP) may cause cell death, resulting in CMS (cytoplasmic male sterility). DNA sequence differences in ATP synthase genes have been revealed between the N- and S-cytoplasms in the cotton CMS system. However, very few data are available at the RNA level. In this study, we compared five ATP synthase genes in the H276A, H276B and fertile F1 (H276A/H268) lines using RNA editing, RNA blotting and quantitative real time-PCR (qRT-PCR) to explore their contribution to CMS. A molecular marker for identifying male sterile cytoplasm (MSC) was also developed.

**Results:**

RNA blotting revealed the absence of any novel *orf* for the ATP synthase gene sequence in the three lines. Forty-one RNA editing sites were identified in the coding sequences. RNA editing showed that proteins had 32.43% higher hydrophobicity and that 39.02% of RNA editing sites had proline converted to leucine. Two new stop codons were detected in *atp6* and *atp9* by RNA editing. Real-time qRT-PCR data showed that the *atp1*, *atp6*, *atp8*, and *atp9* genes had substantially lower expression levels in H276A compared with those in H276B. By contrast, the expression levels of all five genes were increased in F1 (H276A/H268). Moreover, a molecular marker based on a 6-bp deletion upstream of *atp8* in H276A was developed to identify male sterile cytoplasm (MSC) in cotton.

**Conclusions:**

Our data substantially contributes to the understanding of the function of ATP synthase genes in cotton CMS. Therefore, we suggest that ATP synthase genes might be an indirect cause of cotton CMS. Further research is needed to investigate the relationship among ATP synthase genes in cotton CMS.

**Electronic supplementary material:**

The online version of this article (10.1186/s40659-019-0212-0) contains supplementary material, which is available to authorized users.

## Background

Cytoplasmic male sterility (CMS) is a universal and maternally inherited phenomenon in which the male reproductive structure fails to develop. The molecular mechanisms of CMS plants have been extensively studied for several decades [1]. To date, the cause of CMS in several types of plants has been demonstrated to be chimeric open reading frames (ORFs) resulting from rearrangements of the mitochondrial genome. These ORFs include *urf13* of maize CMS-T [1], *orf79* of Boro II rice [2] and *pcf* of petunia [3]. These ORFs are associated with ATP synthase gene promoter regions or portions of coding regions and inhibit the expression of ATP synthase genes [4]. The ATP synthase complex, composed of five subunits encoded by mitochondrial DNA, converts the electrochemical gradient across the inner mitochondrial membrane into ATP for cellular biosynthesis at the terminal step of oxidative phosphorylation [5].

In the cotton CMS system, CMS-D2 and CMS-D8 are derived from the introduction of the cytoplasm of *Gossypium harknessii Brandegee* (D2) and *Gossypium trilobum* (DC) Skovst (D8), respectively, into upland cotton (*Gossypium hirsutum*, AD1) [6, 7]. Cotton CMS was identified more than 40 years ago but has not been employed extensively in hybrid breeding as the cytoplasm of wild species has negative effects on cotton yield [8]. Thus, it is imperative to develop a new system of cotton CMS with cytoplasm from cultivated species. In the present study, a new CMS line, H276A, the cytoplasm of which is derived from cultivated species, was investigated. The cytological differences between the CMS lines and their maintainer lines have been explored [9]; however, the molecular mechanism of CMS remains unknown.

RNA editing is a post-transcriptional process that can synthesize different amino acid sequences from genomic sequences by conversion of cytidine (C) to uridine (U). In plant mitochondrial systems, RNA editing plays an important role in gene expression at the RNA level and is a critical process for generating functional proteins [10]. It has been reported that some types of CMS are associated with inadequate or divergent RNA editing of mitochondrial genes. In CMS-S maize, *orf77* has a similar sequence to *atp9*; however, *orf77* has less RNA editing relative to *atp9*. The novel RNA editing of *orf77* led to CMS by inhibiting the expression of *atp9* [11]. In rice CMS line Yingxiang A, RNA editing caused an amino acid conversion, which produced a non-functional *atp9* subunit [12].

In previous studies of cotton CMS, investigations have focused on the mutation of mitochondrial genome sequences and exploration of the molecular markers of male sterility cytoplasm (MSC). RFLP polymorphisms were detected between CMS-D2 and normal AD1 cytoplasm with probes for *cox1*, *cox2*, and *atp1* [13]. Zhang et al. [14] reported that six genes (*atp1*, *atp9*, *ccmb*, *nad6*, *nad7c* and *rr18*) have RFLPs between P30B and P30A and explored several male sterility cytoplasm markers. In the above studies, several significant RFLPs associated with ATP synthase genes have been revealed between the N- and S-cytoplasm in the cotton CMS system. However, little information is available at the RNA level. In this study, we compared five ATP synthase genes in H276A, H276B and the fertile F1 (H276A/H268) by RNA editing, RNA blotting and qRT-PCR to explore their contribution to CMS. In addition, a molecular marker for identifying MSC was developed.

## Materials and methods

### Plant materials

For gene cloning, qRT-PCR and RNA blotting, three cotton lines were used: CMS (H276A), a maintainer (H276B) and a fertile F1 (H276A/H268). To identify the cytoplasmic characteristics, 37 varieties of cotton were employed including nine CMS lines (NL11-3A, NL11-4A, NL11-5A, NL11-8A, NL11-9A, NL11-21A, NL11-26A, J-1A, and J-4A) with abortive-type H276A cytoplasm; five CMS lines (NC15-43, NC15-41, NC15-39, NC15-37, and NC15-35) with abortive-type Zhong16A [15] cytoplasm; 14 maintainer lines (NL11-3B, NL11-4B, NL11-5A, H11-8B, NH11-9B, NH11-21B, NL11-26B, J-1B, J-4B, NC15-42, NC15-40, NC15-38, NC15-36, and NC15-34); and nine hybrid F1 lines (NL11-3A/H268, NL11-4/H268, NL11-5A/H268, NL11-8A/H268, NL11-9A/H268, NL11-21A/H268, NL11-26A/H268, J-1A/H268, and J-4A/H268). All plant materials were grown in the experimental field of Guangxi University, China under natural conditions.

### Total DNA and RNA extraction

Genomic DNA was extracted from young leaves of each genotype by the CTAB method [16]. Total RNA from anthers at the abortive stage (tetrad stage) was isolated using the Quick RNA Isolation Kit with on-column DNaseI digestion (Huayueyang, China). The integrity and concentration of the nucleic acids were determined using 1% agarose gels and a NanoDrop 2000 (UV spectrometer) (Thermo, USA).

### cDNA synthesis, cloning and sequencing

All primers (Additional file 1) used in this study were designed based on the *Gossypium hirsutum* L. mitochondrial genome (http://www.ncbi.nlm.nih.gov/nuccore/JX065074.1). For each sample, 1 μg total RNA was reverse transcribed into cDNA using TransScript^R^II One-Step gDNA Removal and cDNA Synthesis SuperMix (Trans, China). Elimination of gDNA was verified with primers for *cox2*, which amplified a 1887 bp fragment of the gDNA, including a 1494-bp intron, but a 393-bp fragment was amplified for cDNA. For amplification of ATP synthase genes, 50 ng DNA and 0.5 μl cDNA were used as templates. The amplicons were cloned into the PMD-19T simple vector (TAKARA, Japan), then three gDNA positive clones were selected randomly and sequenced [23].

### qRT-PCR analysis of ATP synthase genes

Relative quantification of five genes in the three cotton lines were conducted by real-time qRT-PCR analysis with a C1000 Touch^TM^ Thermal Cycler (Bio-Rad, USA) with TransStartR Tip Green qPCR SuperMix (Trans, China). A housekeeping gene, *18s*, served as the internal reference. The *18* *s* primer sequences were 5′-ACACTTCACCGGACCATTCAAT/5′-CCTGGAAGAACCCTTTGTGA. The qRT-PCR conditions followed the manufacturer’s procedure: 30 s at 95 °C followed by 42 cycles of heating at 95 °C for 5 s and annealing at 60 °C for 30 s, then heating to 95 °C with an increment of 0.5 °C for 5 s to generate the melt curve. The relative expression level was calculated by the 2−^∆∆^Ct method with three replicates [17].

### RNA editing and gene expression data analysis

The percentage of RNA editing efficiency was determined with at least 13 cDNA clones from each of the three materials (CMS, maintainer line and fertility F1) for each gene [23]. For an RNA editing site, at least two C-U conversions at a position should be detectable to be regarded as an authentic RNA editing site [25]. RNA editing sites are classified as “full editing” when ≥ 80% of recovered sequences contain the converted sequence or “partial editing” when < 80% of recovered sequences contain the converted sequence [18].

### Northern blot analysis

Approximately 30 μg total RNA was denatured and separated on 1% denaturing formaldehyde agarose gels and transferred to a Hybond N+ nylon membrane (GE, UK). The blots were hybridized with a DIG-high labeled cDNA probe at 42 °C for 12 h, and subsequently the manufacturer’s instructions for the DIG-High prime DNA Labeling and Detection Starter Kit II (Roche, Germany) were followed.

### Molecular marker and polyacrylamide gel electrophoresis (PAGE)

The primer sequences of molecular marker were 5′-TACAGGAAGGACTCGCTTTCTCTTT/5′-AAGGCATAACCAGAAGAATTGTGAA. The PCR conditions for the molecular marker were 3 min at 95 °C followed by 42 cycles of heating at 95 °C for 30 s, annealing at 56 °C for 30 s and amplification at 72 °C for 30 s, and a final amplification at 72 °C for 5 min. To analyze the PCR product of the molecular marker, 10% native polyacrylamide gel electrophoresis was used; the 50 ml reagent mix included 12.5 ml 40% polyacrylamide, 40 μl tetramethylethylenediamine (TEMED), 5 ml 10 × tris boric acid (TBE), and 400 μl 10% ammonium persulfate (APS), with ddH2O added to 50 ml. The gel was stained by 10% AgNO_3_ and colored with formaldehyde.

### Statistical analysis

Data on gene expression among CMS, maintainer and fertile lines of cotton were statistically analyzed with one-way ANOVA using SPSS 18.0.

## Results

### Sequence and transcript polymorphisms analysis of five genes

The sequences of five genes (*atp1, atp4, atp6, atp8 and atp9*) in H276A, H276B and a fertile F1 (H276A/H268) were obtained using homology cloning. Sequences analysis (Additional files 2, 3, 4, 5, and 6) revealed no difference in coding regions between CMS line H276A and its maintainer line H276B, but a one base conversion (C > A) at the 171th base of *atp8* resulted in an amino acid change (arginine to serine) at the 56th amino acid. However, a 6-bp deletion in the 5′ flanking region of *atp8* and six base conversions in the untranslated regions (UTR) of *atp1* were identified in H276A in comparison with H276B. In addition, the fertile F1 (H276A/H268) had sequences similar to CMS line H276A for all five genes.

**Fig. 1:**
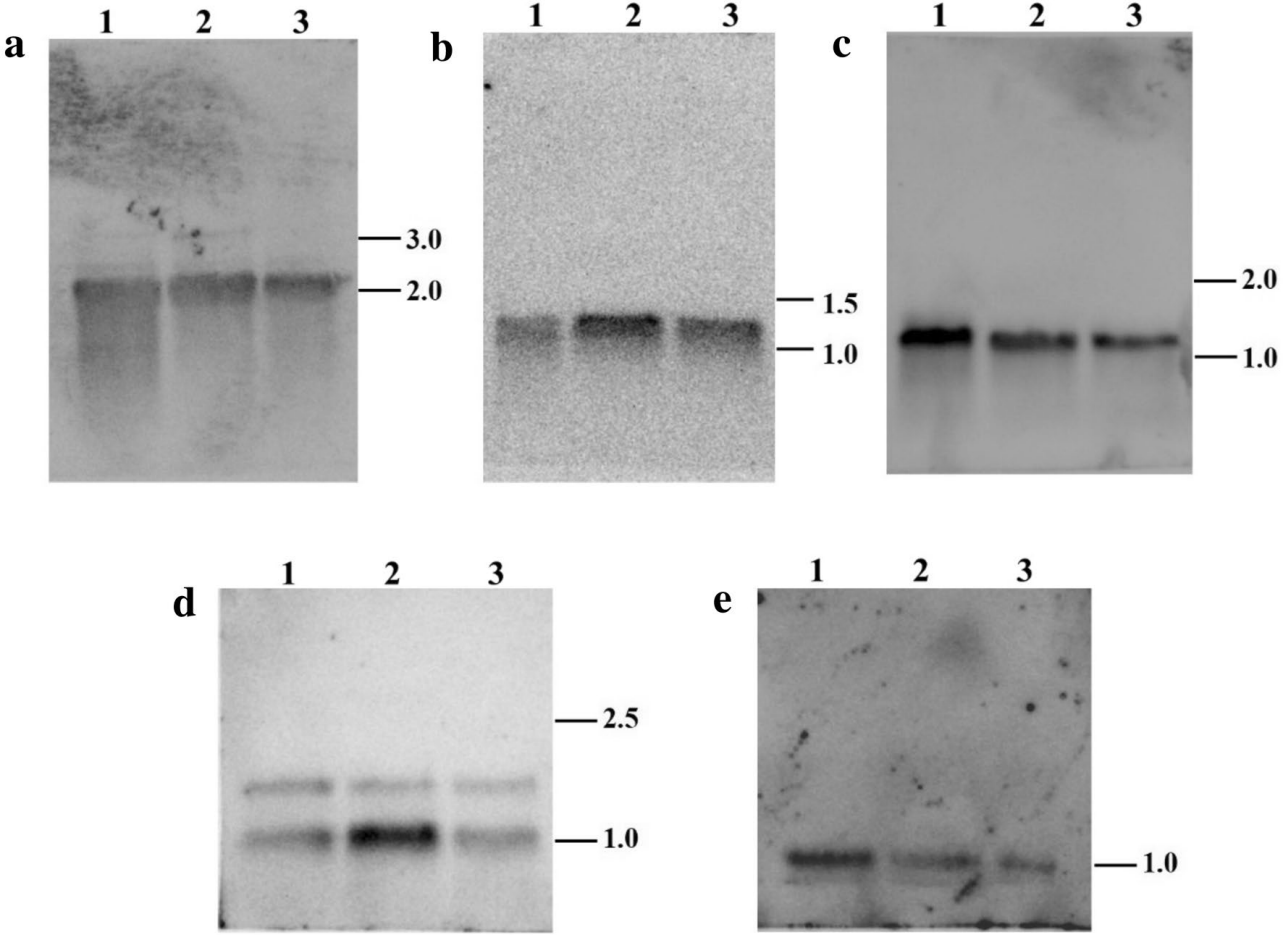
RNA blot of ATP synthase genes. **a** *atp1*; **b** *atp4*; **c** *atp6*; **d** *atp8*; **e** *atp9*; Lane 1: CMS line H276A; Lane 2: Maintainer line H276B; Lane 3: fertile F1 (H276A/H268)

In the present study, northern blotting was used to explore transcript polymorphisms of ATP synthase genes in three cotton materials. The results showed that only one transcript in *atp1*, *atp4*, *atp6* and *atp9*, but two transcripts in *atp8* (Fig. 1). All five genes showed similar transcripts in CMS line H276A, maintainer line H276B and the fertile F1 (H276A/H268). To the best of our knowledge, we are the first to analyze the transcripts of five ATP synthase genes in cotton.

### RNA editing of five ATP synthase genes

**Fig. 2:**
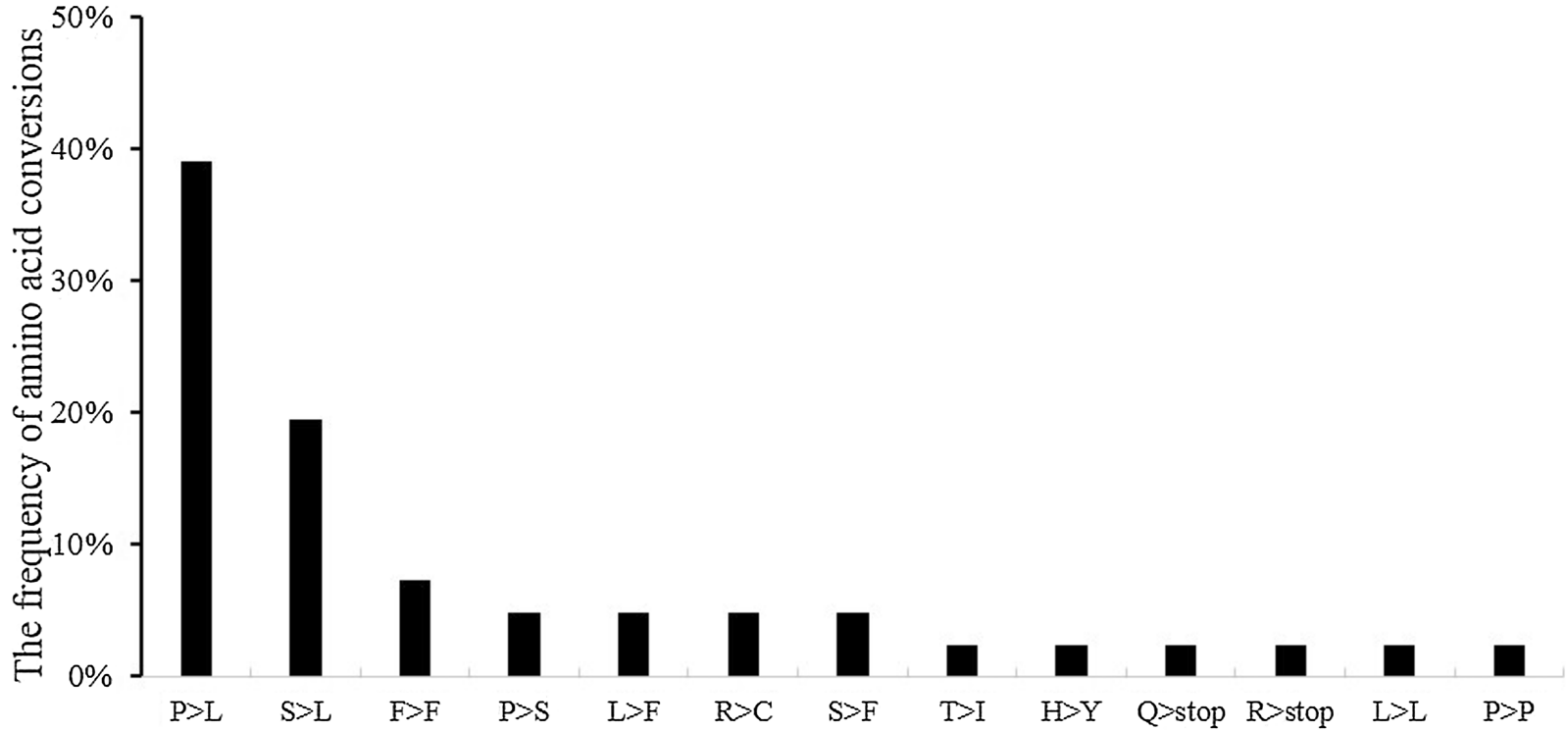
Amino acid conversions resulting from RNA editing events; n = 41

The features and editing frequencies of each editing site of five genes (*atp1*, *atp4*, *atp6*, *atp8* and *atp9*) were detected by sequence cloning (Table 1). A total of 41 editing sites, containing 27 full and 14 partial editing sites, were identified in coding regions. RNA editing results showed that 90.3% of amino acids were changed, and silent RNA editing contributed to 9.75% of the changes. The highest frequencies of amino acid changes were proline (P) > leucine (L) (39.02%), followed by serine (S) > leucine (L) (19.51%), and phenylalanine (F) > phenylalanine (F) (7.32%) (Fig. 2). Amino acid changes led to increased hydrophobic amino acids as described in plant organelles [19]. The frequency of RNA editing sites that resulted in the change from one hydrophobic amino acid to another was 56%. Further, the frequency of hydrophilic to hydrophobic changes was 27%, and a small percentage of changes (4.8%) resulted in hydrophobic to hydrophilic changes. No variation was observed in hydrophilic to hydrophilic amino acid changes (Table 2). The position of RNA editing was mostly at the 2nd codon base (58.54%), which was consistent with findings for *Arabidopsis thaliana* [20], and RNA editing at the 1st and 3rd codon bases was 26.83% and 14.63%, respectively.

**Table 1:** Comparison of the editing sites detected in five mtDNA genes cotton materials

Gene	Position	Code	aa change	Editing efficiency (%)			Codon position
				H276A	H276B	F1(H276A/H268)	
*atp 1*	1039	Ccc	P > S	100	100	100	1
	1064	tCg	S > L	100	100	100	2
	1216	cCt	L > F	100	100	100	2
	1292	cCg	P > L	100	100	100	2
	1415	cCa	P > L	100	100	100	2
	1484	cCa	P > L	100	100	100	2
*atp4*	115	Cgt	R > C	100	90.0	100	1
	212	tCg	S > L	83.3	100	100	2
	221	ttC	F > F	100	100	100	3
	224	cCc	P > L	83.3	100	100	2
	225	ccC	P > L	58.3	90.9	0	3
	245	cCt	P > L	100	100	91.7	2
	247	Ccg	P > L	50	27.2	100	1
	248	cCg	P > L	91.7	100	91.7	2
	392	tCa	S > L	83.3	100	100	2
	404	cCa	P > L	83.3	100	91.7	2
	413	aCt	T > I	83.3	100	91.7	2
	489	ccC	P > P	83.3	90.9	91.7	3
*atp6*	106	Cca	P > S	63.6	63.6	90.9	1
	242	cCg	P > L	54.5	45.5	63.6	2
	294	ttC	F > F	0	18.2	45.5	3
	305	tCg	S > L	45.5	54.5	72.7	2
	323	tCg	S > L	45.5	45.5	45.5	2
	331	Cgt	R > C	63.6	54.5	54.5	1
	338	cCc	P > L	36.4	54.5	63.6	2
	339	ccC	P > L	18.2	45.5	45.5	3
	733	Cat	H > Y	72.7	72.7	81.8	1
	740	tCt	S > F	45.5	63.6	72.7	2
	787	tCt	Q > *	63.6	45.5	81.8	1
*atp 8*	58	Ctc	L > F	100	84.6	100	1
	76	Cca	P > L	100	100	93.3	1
	77	cCa	P > L	100	100	93.3	2
	443	cCa	P > L	100	100	100	2
*atp9*	20	tCa	S > L	100	100	100	2
	50	tCa	S > L	85.7	93.3	100	2
	182	tCg	S > L	100	100	100	2
	191	cCa	P > L	92.6	100	100	2
	205	Ctg	L > L	85.7	86.7	92.9	1
	215	tCc	S > F	100	100	100	2
	223	Cga	R > *	100	100	100	1
	243	ttC	F > F	35.7	66.7	50	3

Position, respect to ATG. Code, capital word represents RNA editing site in code. All the editing forms are C-U conversion

**Table 2:** The frequency of hydrophilic/hydrophobic amino acid changed as a result of RNA editing

Amino acid changed	Number	Frequency (%)
Hydrophobic to hydrophobic	23	62.16
Hydrophilic to hydrophobic	12	32.43
Hydrophobic to hydrophilic	2	5.41
Hydrophilic to hydrophilic	0	0
Total	37	100

Statistic didn’t contain Q > *, R > * and two R > C

### *atp1*, *atp8*

Six and four full editing sites were detected in *atp1* and *atp8*, respectively. However, there was no significant difference noted at any of these sites between CMS line H276A and its maintainer line H276B.

### *atp4*

Twelve editing sites (ten full editing and two partial editing) were identified in *atp4*. The editing frequency of the 225th base, which was located at the 3rd codon base of the 74th amino acid, decreased significantly in H276A. However, the 2nd codon base of the 74th amino acid was a full editing site and had a similar amino acid change (proline to leucine). The editing frequency of the other partial editing site at the 247th base was significantly increased in H276A compared with that of H276B (50% vs. 27.2%).

### *atp6*

A total of eleven partial editing sites were identified in *atp6*. A partial editing site at the 787th base caused a change from glutamine to a stop codon, and the frequency of editing was 63.6% and 45.5% in CMS and the maintainer line, respectively. A novel stop codon was created in the 789 bp coding region though the 816 bp coding region in the wild-type. At the other special editing site at the 294th base, the maintainer line had a significantly higher RNA editing efficiency compared with the CMS line (18.2% vs. 0%).

### *atp9*

Seven full and one partial editing site were found in *atp9*. However, a new stop codon with a similar editing frequency between H276A and H276B was detected. The partial editing site (the 243th base), which had a substantially decreased editing frequency in H276A, was a silent RNA editing site (F > F).

In addition, comparative analysis of the RNA editing frequency between CMS line H276A and the fertile F1(H276A/H268) indicated that, except for *atp4* (225th base), *atp6* (331th base) and *atp8* (77th base), all other RNA editing sites in the F1 had a relatively higher editing efficiency compared with H276A. An increased editing efficiency may be associated with the presence of the restorer gene.

### Relative expression of five genes in anthers of three materials

**Fig. 3:**
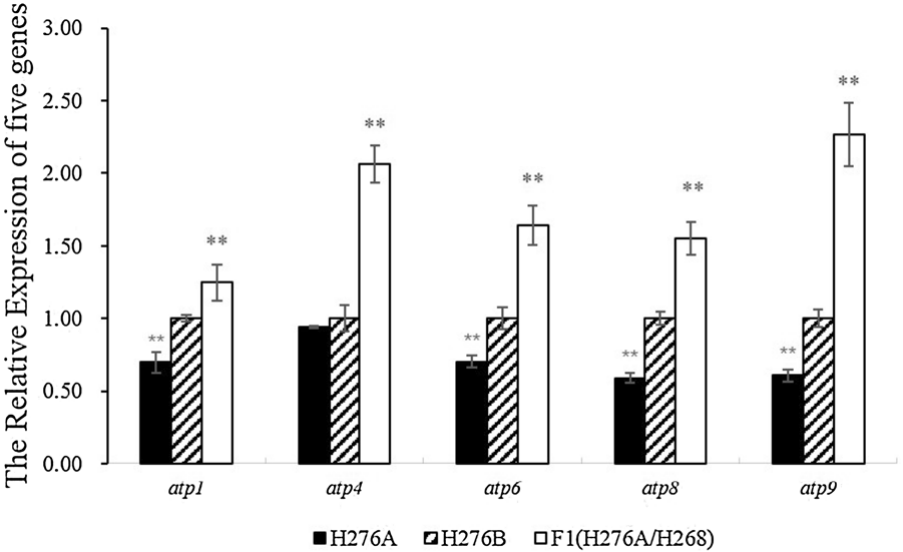
Expression analysis of five genes in cotton anthers by qRT-PCR. The housekeeping gene 18S was used as an internal control; H276A, CMS line; H276B, maintainer line; and H276A/H268, F1 plants from the descendant of restorer H268 hybridized with H276A. Error bars represent standard deviation (n = 3). Double asterisk represents significance at 1% probability

The relative expression levels of five ATP synthase genes in H276A, H276B and a hybrid F1 (H276A/H268) were detected using real-time qRT-PCR. Data were analyzed using SPSS software. The results showed a substantial reduction in the expression levels of *atp1* (0.70), *atp6* (0.70), *atp8* (0.59), and *atp9* (0.61) compared with H276B (P < 0.05). By contrast, a significant increase in the expression levels of *atp1* (1.25), *atp4* (2.06), *atp6* (1.64), *atp8* (1.55) and *atp9* (2.27) in the F1(H276A/H268) relative to the maintainer line H276B (P < 0.01) was observed (Fig. 3).

### Development of a molecular marker to identify MSC based on *atp8*

**Fig. 4:**
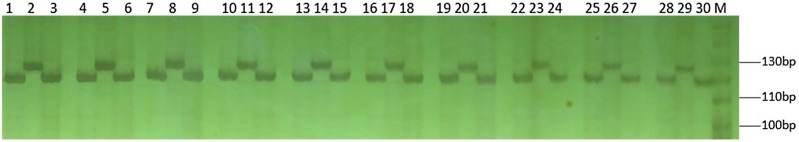
Identification accuracy of the molecular marker. M:10 bp DNA ladder; Lane 1–15: NL11-3A, NL11-3B, NL11-3A/H268, NL11-4A, NL11-4B, NL11-4A/H268, NL11-5A, NL11-5B, NL11-5A/H268, NL11-8A, NL11-8B, NL11-8A/H268, NL11-9A, NL11-9B, and NL11-9A/H268; Lane 16–30: NL11-21A, NL11-21B, NL11-21A/H268, J-1A, J-1B, J-1A/H268, J-4A, J-4B, J-4/H268, NL11-26A, NL11-26B, NL11-26A/H268, H276A, H276B, and H276A/H268

To identify MSC in cotton, a molecular marker based on the 6 bp deletion at the 5′ flanking region of *atp8* in CMS line H276A was developed. The product of special primers BQ*atp8*-F and BQ*atp8*-R was 123 bp and 129 bp in MSC and MFC, respectively, as shown in S.5. Nine cotton CMS lines with abortive type H276A cytoplasm, their maintainer lines and the hybrid F1 were used to detect the accuracy of the molecular marker. A 123 bp fragment was amplified from all CMS lines and their hybrid F1 compared to a 129 bp fragment from their maintainer lines (Fig. 4). The results indicated that the molecular marker could be used to identify MSC in cotton. To extend this observation, five CMS lines with abortive type Zhong16A and their maintainer lines were analyzed, which showed that all CMS lines and all maintainer lines had the same band as in the corresponding CMS line H276A and maintainer line H276B (Additional file 7).

## Discussion

Although there have been consistent findings for ATP synthase genes and CMS in other cotton CMS systems, further research is needed for detailed understanding of the molecular mechanisms of ATP synthase genes at the transcriptional level. To the best of our knowledge, this is the first report to detect CMS-associated genes in cotton using RNA blots. This strategy has been used to identify some critical CMS genes, such as *WA352* in rice [21] and *orf456* in chili pepper [22]. However, our data demonstrated that there was an absence of novel *orf* containing the ATP synthase gene sequence in the CMS line compared with the other two materials.

To gain a detailed understanding of transcription of ATP synthase genes, we investigated their RNA editing, and 41 RNA editing sites were identified. All editing sites had a change from C to U, which corresponds with the observations from rice [23] and CMS-D8 [24]. In other cotton CMS systems with RNA editing sites, there was strong specificity. For instance, *atp1*, *atp4*, *atp6*, *atp8* and *atp9* of CMS-D8 [24] have 7, 9, 15, 2, and 2 editing sites, respectively. However, our study found *atp1*, *atp4*, *atp6*, *atp8* and *atp9* have 6, 12, 11, 4, and 9 RNA editing sites, respectively.

The relationship between CMS and mitochondrial genome RNA editing has been widely studied. Previous studies suggested that silent editing can interrupt normal function and can alter secondary structure of RNA [25]. In the present study, four silent editing sites (three F > F and one P > P) were detected in three materials with similar editing frequencies. In addition, RNA editing may produce a new stop codon that creates a truncated open reading frame. For instance, wheat CMS resulted from novel RNA editing at the 37th base of *atp9* creating a stop codon [10]. In the present study, two new stop codons from RNA editing were identified. Of these, one was located at 223th base of *atp9*, which had not been previously reported in cotton, but no difference existed in the editing frequency of all three materials. The other stop codon was at the 787th base of *atp6*, and its editing frequency increased with fertile recovery. Except for the editing sites located at the 225th base of *atp4* and the 76th and 77th bases of *atp6*, the frequencies of the remaining RNA editing sites increased in the hybrid F1relative to those of H276A. We inferred that it has its own restorer gene. Most restorer genes encode a PPR protein, which is essential for RNA editing of mitochondrial genes [25].

Pollen development is an energy-consuming process, especially during meiosis, and cell death might be caused by low levels of ATP leading to CMS [26]. qRT-PCR of five ATP synthase genes of anthers at the tetrad stage showed that except for *atp4*, the other four genes had remarkably lower expression in H276A; by contrast, all five genes in the hybrid F1 (H276A/H268) were increased dramatically. These results were consistent with *atp8* and *atp9* in kenaf [27, 28], which suggested that restorer genes affect ATP synthase expression. Moreover, these results also suggested that cotton CMS could be associated with decreased expression of ATP synthase genes (*atp1*, *atp6*, *atp8* and *atp9*). However, the relationship between cotton CMS and ATP synthase genes needs further investigation.

Molecular markers have become an important and more convenient tool for identification of MSC in comparison to traditional breeding. In cotton breeding, a shortage of CMS prevented the utilization of cotton heterosis. To overcome this problem, cotton MSC molecular markers were explored based on different sequences. However, to date, all MSC molecules based on PCR were based on mutations around *atp1*, such as scar611 [29] and ssr160 [14].

## Conclusion

We identified no novel *orf* in ATP synthase gene sequences in three cotton lines and 41 RNA editing sites in coding sequences. The result of RNA editing in three materials indicated that no differences were associated with CMS. However, the results of qRT-PCR suggested that ATP synthase genes may be an indirect cause of cotton CMS. The relationship between ATP synthase genes and cotton CMS needs further study.

## Additional files

**Additional file 1.** Primers used in this study.

**Additional file 2.** Sequences analysis of *atp1* in three materials.

**Additional file 3.** Sequences analysis of *atp4* in three materials.

**Additional file 4.** Sequences analysis of *atp6* in three materials.

**Additional file 5.** Sequences analysis of *atp8* in three materials.

**Additional file 6.** Sequences analysis of *atp9* in three materials.

**Additional file 7.** Amplification of molecular marker specific to MSC.

## Abbreviations

CMS: cytoplasmic male sterility
qRT: quantitative reverse transcribed PCR
ATP: adenosine triphosphate
ORFs: open reading frames
MSC: male sterility cytoplasm
MFC: male fertility cytoplasm
