# Supplementary material for: RNA editing analysis of ATP synthase genes in the cotton cytoplasmic male sterile line H276A
Source: Biol Res. 2019 Feb 6;52:6. doi: 10.1186/s40659-019-0212-0 (PMC6364438; doi:10.1186/s40659-019-0212-0)
Supplement: Supplementary file 2 — Additional file 2. Sequences analysis of atp1 in three materials. [file 40659_2019_212_MOESM2_ESM.docx]

**
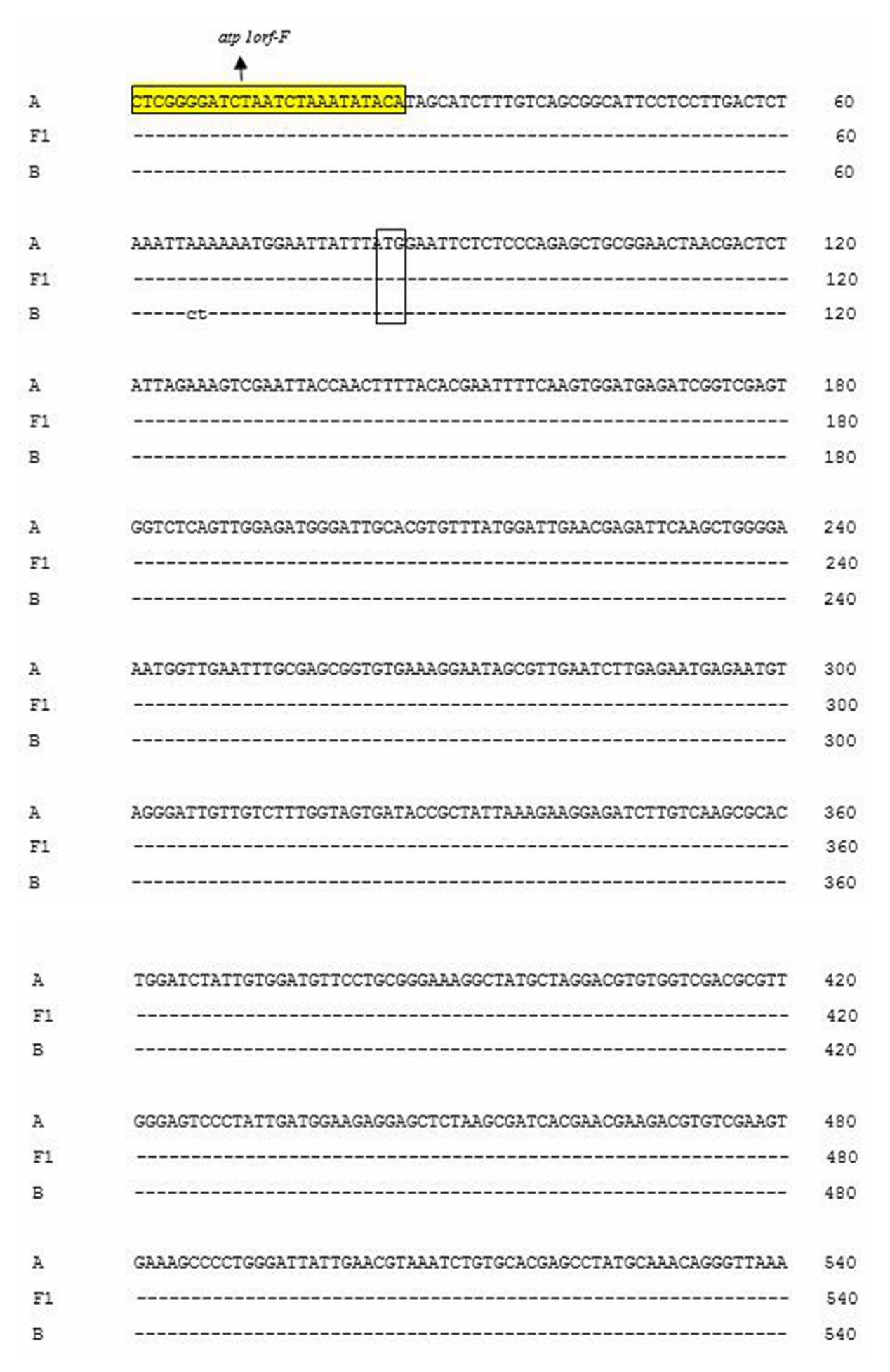
Additional file 2.** Sequences analysis of *atp1* in three materials


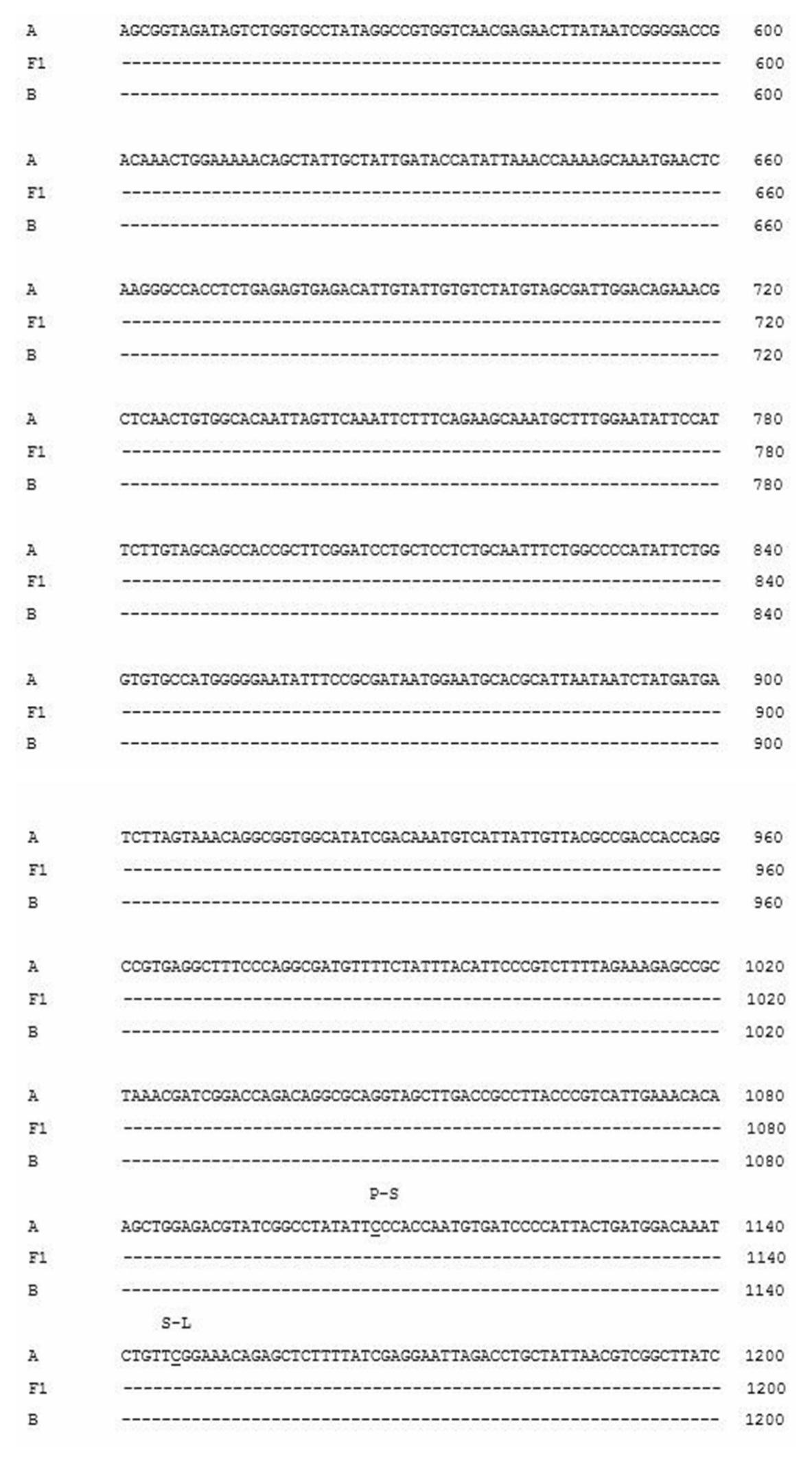


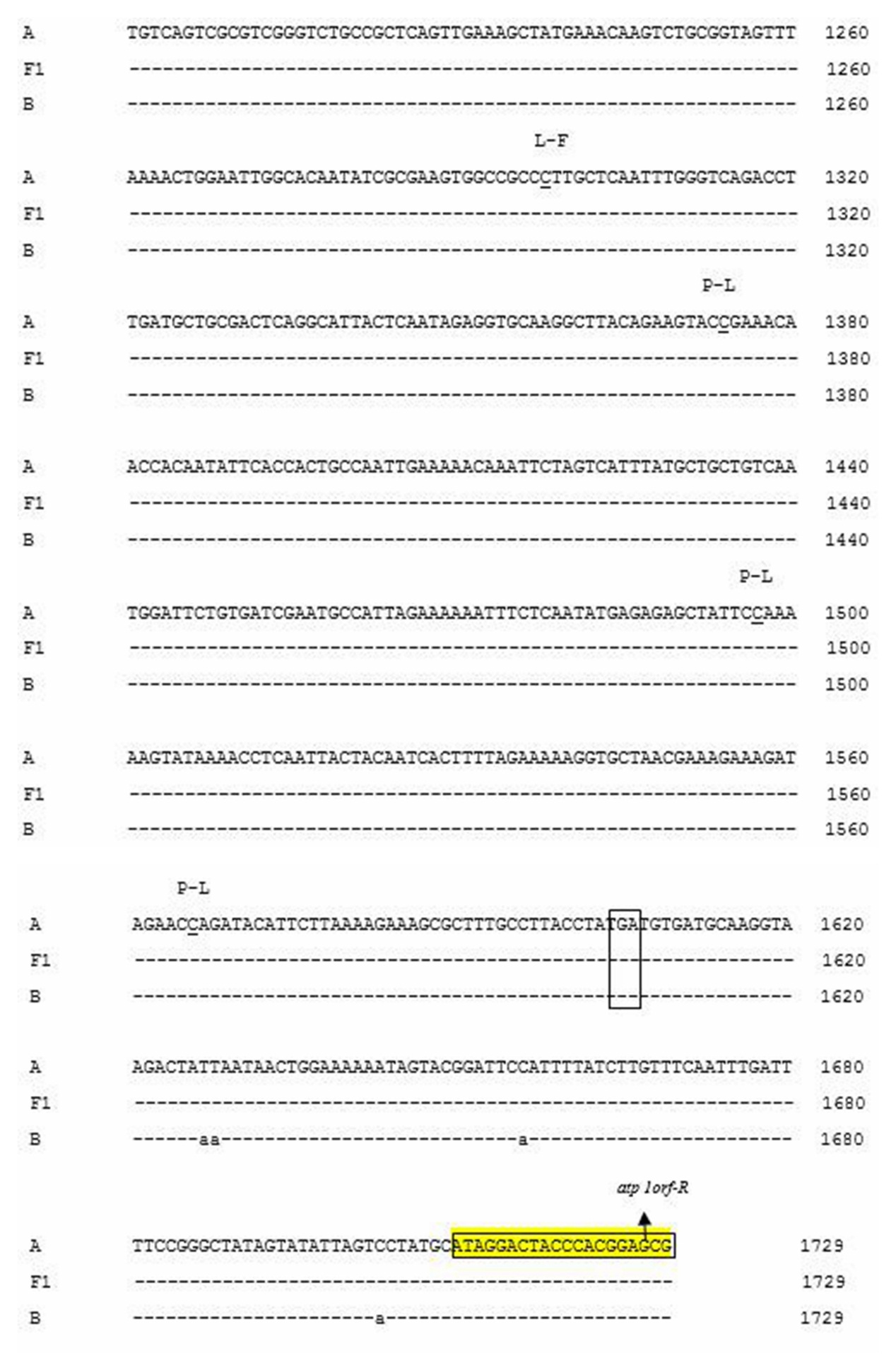
Notes: A, CMS line H276A; F1, Fertile F1 (H276A/H268); B, Maintainer line H276B.
