# Supplementary material for: RNA editing analysis of ATP synthase genes in the cotton cytoplasmic male sterile line H276A
Source: Biol Res. 2019 Feb 6;52:6. doi: 10.1186/s40659-019-0212-0 (PMC6364438; doi:10.1186/s40659-019-0212-0)
Supplement: Supplementary file 5 — Additional file 5. Sequences analysis of atp8 in three materials. [file 40659_2019_212_MOESM5_ESM.docx]

**Additional file 5.** Sequences analysis of *atp8* in three materials


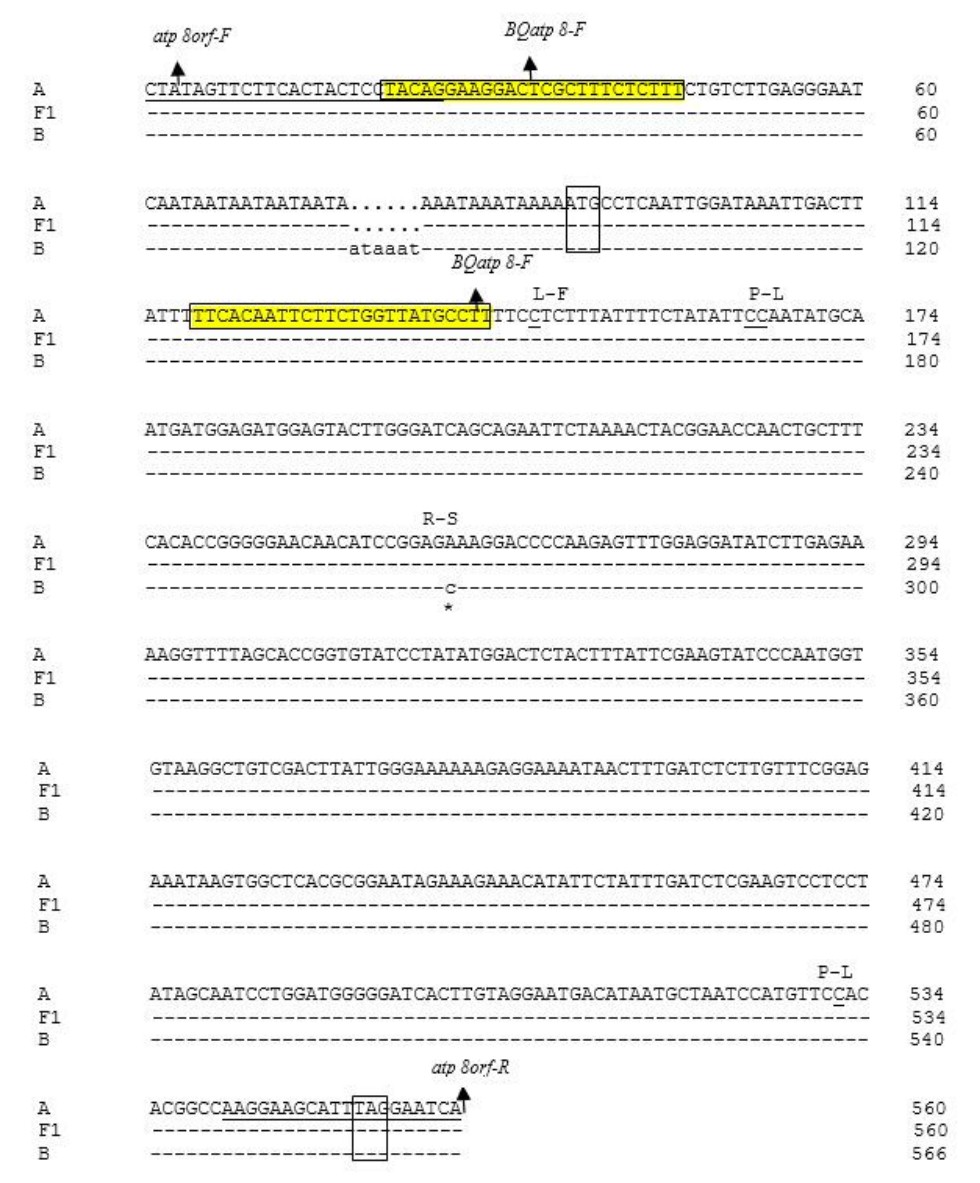


Notes: A, CMS line H276A; F1, fertile F1 (H276A/H268); B, Maintainer line H276B.
