# Supplementary material for: RNA editing analysis of ATP synthase genes in the cotton cytoplasmic male sterile line H276A
Source: Biol Res. 2019 Feb 6;52:6. doi: 10.1186/s40659-019-0212-0 (PMC6364438; doi:10.1186/s40659-019-0212-0)
Supplement: Supplementary file 7 — Additional file 7. Amplification of molecular marker specific to MSC. [file 40659_2019_212_MOESM7_ESM.pdf]

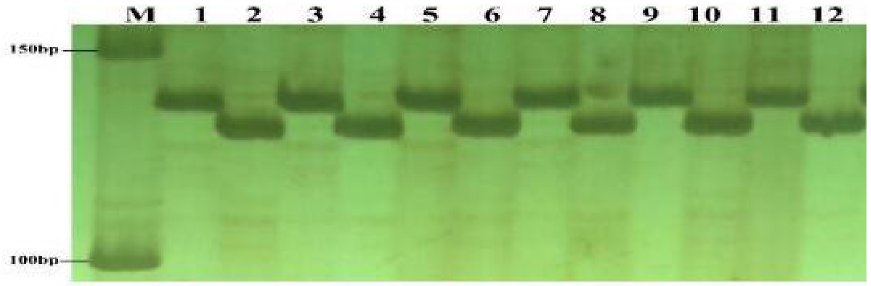

Additional file 7. Amplification of molecular marker specific to MSC.
M: 50bp DNA ladder; Lane 1-12: H276B, H276A, NC15-43, NC15-42, NC15-41, NC-15-40, NC15-39, NC15-38, NC15-37, NC15-36, NC15-35, NC15-34.
